# Supplementary material for: Clustering method for time-series images using quantum-inspired digital annealer technology
Source: Commun Eng. 2024 Jan 10;3:10. doi: 10.1038/s44172-023-00158-0 (PMC10955962; doi:10.1038/s44172-023-00158-0)
Supplement: Supplementary file 2 — Supplementary information [file 44172_2023_158_MOESM2_ESM.pdf]

Supplementary Information

Clustering Method for Time-Series Images Using Quantum-Inspired Digital Annealer Technology

Tomoki Inoue[1], Koyo Kubota[1], Tsubasa Ikami[2], Yasuhiro Egami[3], Hiroki Nagai[2], Takahiro Kashikawa[4], Koichi Kimura[4], Yu Matsuda[1, *]

1. Department of Modern Mechanical Engineering, Waseda University, 3-4-1 Ookubo, Shinjuku-ku, Tokyo, 169-8555, Japan
2. Institute of Fluid Science, Tohoku University, 2-1-1 Katahira, Aoba-ku, Sendai, Miyagi-prefecture 980-8577, Japan
3. Department of Mechanical Engineering, Aichi Institute of Technology, 1247 Yachigusa, Yakusa-Cho, Toyota, Aichi-prefecture 470-0392, Japan
4. Quantum Application Core Project, Quantum Laboratory, Fujitsu Research, Fujistu Ltd, Kawasaki, Kanagawa 211-8588, Japan

* corresponding author: Yu Matsuda

Supplementary Note 1: Clustering of online available datasets

To further investigate the proposed method, we applied the proposed method to classify the "Fungi" and "Mallat" datasets available from the UEA & UCR time-series classification repository. [1-3] The "Fungi" dataset contains eighteen clusters and high-resolution melt curves of the rDNA internal transcribed spacer region. The calculation conditions were the same as those in the main text. Two typical clustering results are shown in Fig. S1. As in the main text, ensemble-averaged data for each method were calculated and are shown in Fig. S1. The dataset was also classified using the "TimeSeriesKMeans" function in "tslearn." [4] We compare the root mean squared error (RMSE) between the ensemble-averaged data of the correct data and those obtained using the proposed method and "tslearn". The RMSEs of the proposed and existing methods shown in Fig. S1(a) were 0.00 and 0.09, respectively. The proposed method correctly classified the data points into this class. The RMSEs of the proposed and the existing methods shown in Fig. S1(b) were 0.14 and 0.00, respectively. Here, the existing method showed better results. The classification performance of the two method is considered comparable.

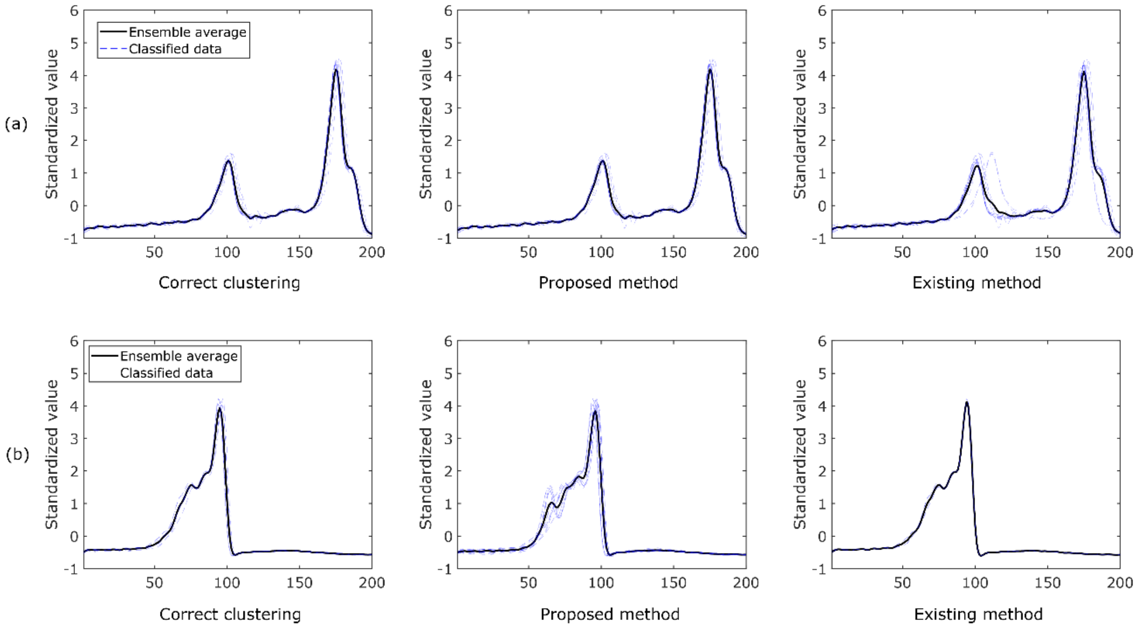

Figure S1. Typical clustering results for "Fungi" dataset from the UEA & UCR time-series classification repository using the proposed and existing methods. The data labeled as class 2 and class 10 in the repository are shown in (a) and (b), respectively.

The “Mallat” dataset is a simulated dataset that contains eight clusters.[3] This dataset was also classified using the proposed method and “tslearn.” Two typical results are also shown in Fig. S2. The RMSEs of the proposed and existing methods shown in Fig. S2(a) were 0.00 and 0.00, respectively. The RMSEs of the proposed and existing methods shown in Fig. S2(b) were 5.0×10^{-3} and 0.09, respectively. Both methods correctly classified the data.

These results further confirm that the performance of the proposed method is comparable to that of the existing method for these datasets.

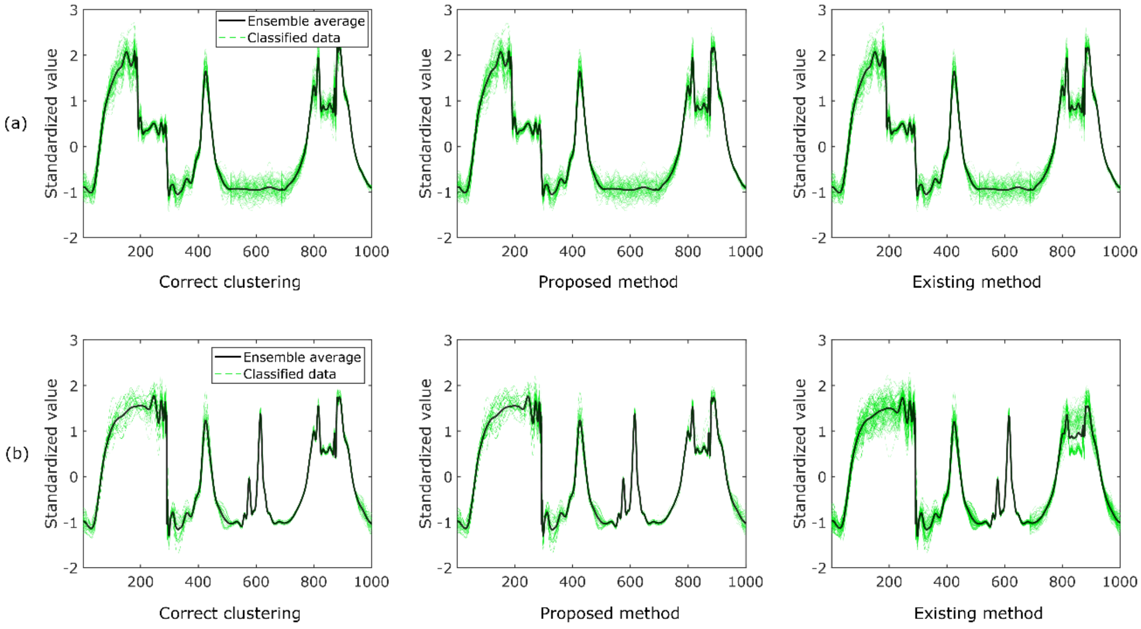

Figure S2. Typical clustering results for “Mallat” dataset from the UEA & UCR time-series classification repository using the proposed and existing methods. The data labeled as class 2 and class 3 in the repository are shown in (a) and (b), respectively.

Supplementary Note 2: Effect of parameter for clustering

We investigated the effect of λ_2 in Eq. (4) of the main text on the clustering performance. The parameter λ_2 adjusts the number of data points in each cluster. In the main text, we set $\lambda_2 = \lambda_1/40$ for the clustering calculation of the flow measurement dataset. We classified the dataset with $\lambda_2 = 0$ and $\lambda_2 = \lambda_1/67$. The results are shown in Fig. S3. The number of clusters was reduced to three when $\lambda_2 = 0$ (Fig. S3(a)). Moreover, we recognized that the outliers are also classified the clusters. When $\lambda_2 = \lambda_1/67$, overlaps of the clusters were observed. Some of outliers were not classified into the clusters. These results are caused by the small penalty term for the number of data points to be classified into each cluster. When $\lambda_2 = \lambda_1/30$ (Fig. S3(c)) and $\lambda_2 = \lambda_1/20$ (Fig. S3(d)), the outliers, which are the datapoints outside the circle with a radius of 1/2, were not classified into any clusters. However, there are many datapoints on the circle that were not classified into the clusters. As λ_2 increases, the number of data points classified into clusters becomes equal and smaller.

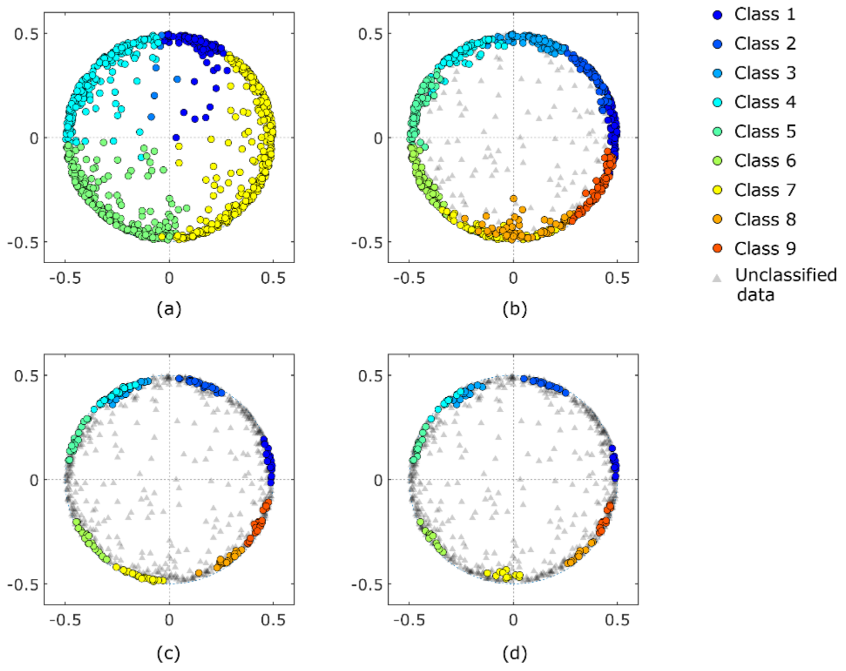

Figure S3. Clustering results shown in two-dimensional scatter plot based on MDS. (a) $\lambda_2 = 0$, (b) $\lambda_2 = \lambda_1/67$, (c) $\lambda_2 = \lambda_1/30$ and (d) $\lambda_2 = \lambda_1/20$, where λ_1 and λ_2 are the parameter of Eq. (4) in the main text.

Supplementary Note 3: Code for time-series clustering

The code for the time-series clustering developed in this study is shown. The code was executed in the third-generation Fujitsu Digital Annealer (DA3).

Clustering.py

```
# Clustering algorithm using DA3
import numpy as np
from pprint import pprint
from pyqubo import Array, Constraint, Placeholder, solve_qubo

import re
from pq2da3 import *
import json
import codecs
import pandas as pd
import time, copy, collections, random
from python_fjda import fujitsu_json, fjda_scheme, fjda_client
import sys
import gc

def load_matrix(filename):

    lines = open(filename).readlines()

    n_cluster = int(lines[0].split()[0])
    n_edge = int(lines[0].split()[1])

    d = []
    for i in range(n_edge):
        parts = (lines[i + 1].split())
        parts2 = []
        for j in range(n_edge):
            a = int(parts[j])
            parts2.append(a)
        d.append(parts2)
        # if dist <= criteria and a < b:
        #       dist = 1
        #       #edges.append((a, b, c))
        #       edges.append((a, b))

    # d = [list(map(int, input().split())) for i in range(n_edge)]

    return n_cluster, n_edge, d

dac = fjda_client.fjda('dau2-05-1.da.labs.fujitsu.com')

n_cluster, n_edge, d = load_matrix('simil2_1to5_50.txt')
```

```
print((d))

print('cluster_num:',n_cluster)
print('edge_num:',n_edge)

# number
q = Array.create('q', shape=(n_cluster,n_edge), vartype='BINARY')
print("1")

t = n_edge
k = n_cluster
H_A = sum(sum(d[i][j]*q[c,j]*q[c,i] for j in range(t) for i in range(t) if j != i) for c in range(k))
print("2")
H_B = Constraint(sum((1-sum(q[c,j] for c in range(k)))**2 for j in range(t)),'HB') # constraint1
print("3")
H_C = Constraint(sum(sum(q[i,j] for j in range(t))**2 for i in range(k)),'HC') # constraint2

print("3-2")
lambda_B = 1000000
lambda_C = 25000

H = -(H_A)+lambda_B*H_B+lambda_C*H_C

model = H.compile()
print("4")
del q, d, H_A, H_B, H_C
del H

gc.collect()

qubo, offset = model.to_qubo(index_label = True)
fj_data = da3_pra(qubo, offset)
print("5")


false = False    # handling boolean value, JSON --> Python
true = True

fj_qubo_request = fujitsu_json.QuboRequest_fromdict(fj_data)
fj_qubo_request.fujitsuDA3.time_limit_sec = 60
fj_qubo_request.fujitsuDA3.num_output_solution    = 1
fj_qubo_request.fujitsuDA3.seed=123
#fj_qubo_request.fujitsuDA3.penalty_coef = 10000

if 1:
    qs = dac.qstat()
    qa = dac.qstatAsync()
```

```
    print('Use fjda-server', dac.fjda_server)
    print(len(qs.data), 'job(s) in synchronous queue.')
    print(len(qa), 'job(s) in asynchronous queue.')


fj_res = dac.v3_qubo_solve(basic_request=fj_qubo_request) # submit job
status = fj_res.status

while True:
    print('Waiting for', fj_res.id, status)
    #    On bash command line, you can delete job, like this.
    #    python -m python_fjda.fjda_client --fjda-server=dau2-05-0.da.labs.fujitsu.com fjda-server
delete_job --job-id ea5fd79c99244255a442b0b7a0746e33
    time.sleep(10)
    fj_res2 = dac.get_results(fj_res.id)
    status = fj_res2.status
    if status in ('completed', 'deleted', 'error'):
        break

r = fj_res2.results

print('_api_time:', r._api_time)
df = pd.DataFrame({'state': list(r.state_min),
                   'cost':    r.cost_min,
                   'penlty': r.penalty_min}).sort_values(by='cost')
print(df)


def show_res2(result):
    ene = []
    freq = []
    qbit_on = []
    j = 0
    k = 0

    global df_tmp

    state_min_o_n = result['state'].tolist()

    for tmp in state_min_o_n:
        qbit = []
        for i in range(len(tmp)):
            if tmp[i] == 1:
                qbit.append(i)
        flg_append = 1
        for i in range(j):
            if qbit == qbit_on[i]:
                freq[i] += 1
```

```
                flg_append = 0
                break
        if flg_append == 1:
            ene.append(result['cost'][k])
            freq.append(1)
            qbit_on.append(qbit)
            j += 1
        k += 1
    print(ene)
    result2 = {'Energy': ene,'Freq': freq,'Qbit_On': qbit_on}
    df_tmp = pd.DataFrame(result2)
    df_sort = df_tmp.sort_values('Freq', ascending=False)
    df_tmp = df_sort.reset_index(drop=True)
    df_sort = df_tmp.sort_values('Energy', ascending=True)
    df = df_sort.reset_index(drop=True)
    pd.options.display.max_colwidth = 160
    return df, qbit_on

ans1, ans2 = show_res2(df)


filename = '{0}.txt'.format(n_cluster)
f = open(filename,'w')

for data in ans2:
    # show_res3(data)
    f.write("nonzero_num:%s " % len(data))
    # f.write("%s¥n" % data)
    a=[0]*n_edge
    b=[[]]*n_edge
    d=[0]*n_cluster
    for j in data:
        a[j%n_edge] = j//n_edge+1
        b[j%n_edge] = b[j%n_edge]+[j//n_edge+1]
        d[j//n_edge]+=1

    f.write("edge_num:%s¥n" % sum([i>0 for i in a]))

    for j in range(n_cluster):
        f.write("%s " % a.count(j+1))
    f.write("¥n%s¥n" % a)

    f.write("clu:%s¥n" % d)

    c = [0]*n_edge
    for j in range(n_edge):
        c[j] = len(b[j])
    f.write("length:%s¥n" % c)
```

```
    f.write("%s¥n" % b)
    # f.write("%s " %data)


    print("H1",)
    print("H2",)
f.close()

df.to_csv('freq_result.txt')
```

pq2da3.py

```
## QUBO for DA3

from typing import Any, Dict, List, Optional, Union

TypeNumber = Union[int, float]

def int_if_possible(value: TypeNumber) -> TypeNumber:
    """Convert to int if it is the same value."""
    int_value = int(value)
    if int_value == value:
        value = int_value
    return value

def da3_pra(qubo,offset):
    terms = []
    for (index1, index2), coefficient in qubo.items():
        assert isinstance(index1, int) and isinstance(index2, int)
        terms.append((coefficient, [index1, index2]))

    q1 =    [{'coefficient': int(term[0]), 'polynomials': term[1]} for term in terms]

    if offset != 0:
        q1.append({'coefficient': int(offset), 'polynomials': []})

    da_qubo = {'terms': q1}

    q = {}
    q['binary_polynomial'] = da_qubo

    da_para = {
    "fujitsuDA3": {
    "time_limit_sec": 1,
    "num_run":16
    }}
```

```
fj_data = dict(**da_para,**q)

return fj_data
```

Supplementary References

1 Bagnall, A., Lines, J., Vickers, W. & Keogh, E. *The UEA & UCR Time Series Classification Repository*, <www.timeseriesclassification.com> (2017).

2 Lu, S. *et al.* Dynamic time warping assessment of high-resolution melt curves provides a robust metric for fungal identification. *PLOS ONE* **12**, e0173320 (2017). https://doi.org/10.1371/journal.pone.0173320

3 Mallat, S. *A Wavelet Tour of Signal Processing*. (Elsevier Science, 1999).

4 Romain Tavenard, J. F., Gilles Vandewiele, Felix Divo, Guillaume Androz, Chester Holtz, Marie Payne, Roman Yurchak, Marc Rußwurm, Kushal Kolar, Eli Wood. Tslearn, A Machine Learning Toolkit for Time Series Data. *Journal of Machine Learning Research* **21**, 1--6 (2020).
